# Magnitude and time course of insulin resistance accumulation with the risk of cardiovascular disease: an 11-years cohort study

**DOI:** 10.1186/s12933-023-02073-2

**Published:** 2023-12-13

**Authors:** Xue Tian, Shuohua Chen, Qin Xu, Xue Xia, Yijun Zhang, Penglian Wang, Shouling Wu, Anxin Wang

**Affiliations:** 1 Department of Neurology, Beijing Tiantan Hospital, Capital Medical University, Beijing, China; 2 China National Clinical Research Center for Neurological Diseases, Beijing Tiantan Hospital, Capital Medical University, No.119 South 4th Ring West Road, Fengtai District, Beijing, 100070 China; 3 Department of Epidemiology and Health Statistics, School of Public Health, Capital Medical University, Beijing, China; 4 Beijing Municipal Key Laboratory of Clinical Epidemiology, Beijing, China; 5 Department of Cardiology, Kailuan Hospital, North China University of Science and Technology, 57 Xinhua East Rd, Tangshan, 063000 China; 6 Department of Clinical Epidemiology and Clinical Trial, Capital Medical University, Beijing, China

**Keywords:** Cardiovascular disease, Metabolic score for insulin resistance, Time course analysis, Prospective cohort study

## Abstract

**Background:**

The risk of cardiovascular disease (CVD) depended on the magnitude and exposure duration of insulin resistance (IR). This study aimed to investigate the associations of cumulative metabolic score for IR (cumMETS-IR) with incident CVD, and to further explore the modulated effects of time course of METS-IR accumulation.

**Methods:**

We enrolled 47,270 participants without CVD and underwent three examinations during 2006–2010 from the Kailuan study. CumMETS-IR from 2006 to 2010 were calculated as the mean values of METS-IR between consecutive examinations multiplying by time intervals between visits. Time course of METS-IR accumulation was calculated as the slope of METS-IR versus time. Hazard ratios (HRs) and 95% confidence intervals (CIs) for CVD risk were calculated with multivariable-adjusted Cox regressions.

**Results:**

During a median follow-up of 10.99 years, we identified 3184 cases of incident CVD. The risk of incident CVD increased with increasing cumMETS-IR (HR, 1.77; 95% CI 1.58–1.98 for the Q4 versus Q1 group), exposure duration (HR, 1.60; 95% CI 1.45–1.77 for 6 years versus 0 years), and cumulative burden (HR, 1.49; 95% CI 1.37–1.61 for burden ≥ 0 versus < 0). A positive slope was associated with 14% higher risk of CVD (HR, 1.14; 95% CI 1.07–1.22). When combining cumMETS-IR and slope, those with cumMETS-IR ≥ median (142.78) and slope ≥ 0 had the highest risk of CVD (HR,1.38; 95% CI 1.25–1.53).

**Conclusions:**

The risk of CVD increased with elevated cumMETS-IR and an increasing trend over time, emphasizing the importance of maintaining optimal METS-IR levels across life span.

**Supplementary Information:**

The online version contains supplementary material available at 10.1186/s12933-023-02073-2.

## Introduction

Insulin resistance (IR), which refers to the diminished or impaired insulin sensitivity of target organs or tissues shown as impairments in absorbing and oxidizing the glucose [1, 2], has been confirmed as an important predisposing factor in many chronic diseases [3–6]. Although the gold-standard method for assessing IR was the hyperinsulinemic-euglycemic clamp, it may be a great challenge for daily clinical application of this index due to the complex, time-consuming, and resource-consuming shortcomings [7]. Recently, several alternative non-insulin-based measures of IR combined simple routine biochemical indicators, such as the ratio of triglyceride to high-density lipoprotein cholesterol (TG/HDL) and triglyceride glucose index (TyG), have been developed [8, 9]. Whereas, these indices ignore the role of nutritional status in insulin sensitivity. Giving the limitations, the metabolic score for insulin resistance (METS-IR) index has been emerged as another alternative measure of IR, which represented nutritional status and showed a higher concordance with the gold-standard in assessing IR. Additionally, the METS-IR has been reported to have better diagnostic efficacy than the TG/HDL and TyG index [10].

Considering the practicality of its measurements and the pathophysiological correlations with components of metabolic syndrome and IR, a predictive role of METS-IR has also been highlighted in endothelial dysfunction and inflammatory [11, 12]. Moreover, accumulative evidence suggested that METS-IR was related to cardiometabolic disorders and cardiovascular diseases (CVDs) [13–18]. Nevertheless, an inherent limitation of these previous studies is that the METS-IR was evaluated at a single time point. To our knowledge, the components of the METS-IR were affected by many biological and environmental factors, a single measurement of a high METS-IR does not indicate that the body state has experienced a high METS-IR for a long time, which may lead to misclassification of risk assessment of CVD. Capturing both the exposure intensity and the duration, and incorporating cumulative exposure and the time course of the accumulation may provide additional information for the risk assessment of CVD.

Therefore, based on a large cohort study, we aimed to quantify the association of (1) cumulative METS-IR (cumMETS-IR); (2) exposure duration of high METS-IR; (3) cumulative burden of METS-IR with the risk of CVD, and further to assess whether the associations were modulated by time course of METS-IR accumulation.

## Methods

### Study population

**Fig. 1:**
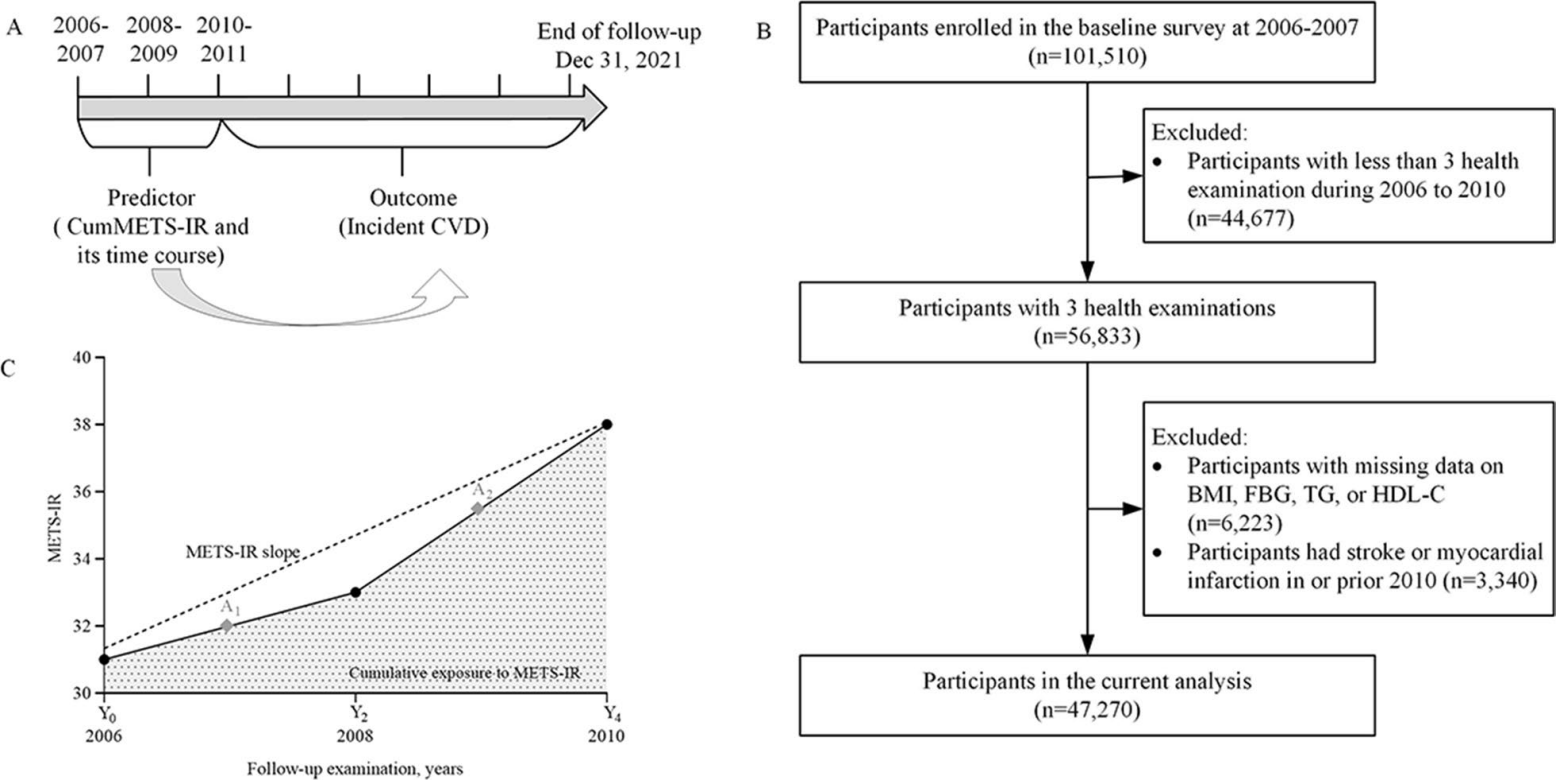
Design of the study **A**. Time line of the study **B**. The flowchart of the study C. Illustration of cumulative and time course of METS-IR over time *BMI* body mass index, *FBG* fasting blood glucose, *HDL-C* high density lipoprotein cholesterol, *METS-IR* metabolic score of insulin resistance, *TG* triglyceride

The participants were recruited from the Kailuan study, which was an ongoing prospective cohort study conducted in Tangshan, China. Details of the study design and procedure have been described previously [19–21]. From June 2006 to October 2007, a total of 101,510 participants aged 18–98 years were enrolled in the baseline survey. They underwent questionnaire assessments, physical examinations, laboratory tests, and then were followed up biennially until 31 December 2021. In the present study, cumMETS-IR was developed during 2006–2010 to predict incident CVD risk from 2010 to 2021 (Fig. 1A). We excluded participants with less than three physical examinations, with missing data on components of METS-IR, and a history of CVD or who died in or prior to 2010. Terminally, a total of 47,270 participants were enrolled (Fig. 1B). The study was performed according to the guidelines of the Declaration of Helsinki and was approved by the Ethics Committee of Kailuan General Hospital (approval number: 2006–05). All participants provided written informed consent.

### Data collection

Information on demographic characteristics, lifestyle, and medical history was collected though face-to-face interview via a standard questionnaire. Height, weight, and blood pressure were measured by professionally trained doctors. Body mass index (BMI) was calculated as weight divided by height squared (kg/m^2^). Fasting blood samples were collected in the morning after an 8- to 12 h overnight fast. All the.

plasma samples were assessed using an auto-analyzer (Hitachi 747, Tokyo, Japan) at the central laboratory of Kailuan Hospital, including fasting blood glucose (FBG), lipid profiles (total cholesterol, triglyceride [TG], low-density lipoprotein cholesterol, and high-density lipoprotein cholesterol [HDL-C]), serum creatinine, and high sensitivity C reactive protein (hs-CRP). Estimated glomerular filtration rate (eGFR) was calculated using Chronic Kidney Disease Epidemiology Collaboration creatinine Eq. 22

### Cumulative METS-IR and its time course

The equation for METS-IR calculation was as follows [10]:

$${\text{METS}} - {\text{IR}} = {{({\text{Ln(2*FBG + TG)}}\,{\text{* BMI}}} \mathord{\left/ {\vphantom {{({\text{Ln(2*FBG + TG)}}\,{\text{* BMI}}} {({\text{Ln}}}}} \right. \kern-0pt} {({\text{Ln}}}}\,({\text{HDL - C)}}$$

CumMETS-IR was defined as the summed average METS-IR for each pair of consecutive examinations multiplied by the time between these two consecutive visits in years:

$${\text{cumMETS}} - {\text{IR}} = \,[{{({\text{METS}} - {\text{IR}}_{{{2}00{6}}} + {\text{METS}} - {\text{IR}}_{{{2}008}} )} \mathord{\left/ {\vphantom {{({\text{METS}} - {\text{IR}}_{{{2}00{6}}} + {\text{METS}} - {\text{IR}}_{{{2}008}} )} {2 \times }}} \right. \kern-0pt} {2 \times }}{\text{time}}_{{{2}00{6} - {2}00{8}}} ] + {{[({\text{METS}} - {\text{IR}}_{{{2}00{8}}} + {\text{METS}} - {\text{IR}}_{{{2}0{1}0}} )} \mathord{\left/ {\vphantom {{[({\text{METS}} - {\text{IR}}_{{{2}00{8}}} + {\text{METS}} - {\text{IR}}_{{{2}0{1}0}} )} {{2}\, \times \,{\text{time}}_{{{2}00{8} - {2}0{1}0}} ]}}} \right. \kern-0pt} {{2}\, \times \,{\text{time}}_{{{2}00{8} - {2}0{1}0}} ]}}$$

Where METS-IR _2006_, METS-IR _2008_, METS-IR _2010_ indicated METS-IR at baseline, the second examinations (2008), and the third examination (2010), time_2006-2008_ and time_2008-2010_ indicated the participant-specific time interval between consecutive examinations in years (Fig. 1C). High METS-IR exposure duration was defined as the times of visits with a high METS-IR (over the cutoff mentioned in the Statistical analysis) among the 3 visits, quantified as 0 year, 2 years, 4 years, and 6 years. Cumulative burden of METS-IR was calculated as [(METS-IR _2006_ + METS-IR _2008_)/2–cutoff] × time_2006-2008_ + [(METS-IR _2008_ + METS-IR _2010_)/2-cutoff] × time_2008-2010_. If the values of cumulative burden were less than 0, this value would be considered as 0.

Time course of cumMETS-IR accumulation was calculated as a slope of METS-IR over time from 2006 to 2010 using the linear regression and the least-squares principle, where METS-IR was taken as the dependent variable, and time from 2006 to 2010 as the independent variable, with a positive or negative slope indicating an increase or decrease in METS-IR over time (Fig. 1C). Change patterns of METS-IR at the three time points were classified into decrease-decrease, decrease-increase, increase–decrease, and increase-increase.

### Assessment of outcomes

Participants were followed up via face-to-face interviews at every 2 year routine medical examination until event of interest, death, or the end of the follow-up (December 31, 2021). The primary outcome in the study was incident CVD, including incident stroke and myocardial infarction (MI). We used ICD-10th revision codes to identify CVD cases (I21 for MI, I60 to I61, and I63 for stroke). All participants were linked to the Municipal Social Insurance Institution and the Hospital Discharge Register for incidence of CVD, which cover all of the Kailuan study participants and updated annually during the follow-up period. To further identify potential CVD events, we reviewed the discharge lists from the 11 hospitals during 2006–2021 and asked for a history of CVD via a questionnaire during the biennial interview. For all suspected CVD events, 3 experienced physician adjudicators who were blinded to the study design reviewed the medical records. The diagnosis of incident stroke was confirmed by medical review, using the World Health Organization criteria [23]. MI was diagnosed according to the criteria of the World Health Organization based on the clinical symptoms, changes in the serum concentrations of cardiac enzymes and biomarkers, and electrocardiographic results [24].

### Statistical analysis

Participants were classified according to quartiles of cumMETS-IR, METS-IR slope (positive or negative), or the combination of median cumMETS-IR with slope, respectively. The optimal cutoff point for METS-IR associated with incident CVD was determined using an outcome-oriented method to maximized log-rank statistics [25].

Baseline characteristics were compared using student t test, analysis of variance, Wilcoxon, or the Kruskal–Wallis test according to distribution, and categorical variables were compared with chi-square test. Kaplan–Meier curves were used to estimate the cumulative incidence of CVD and the differences in curves were compared with the log-rank test.

Multivariable-adjusted Cox proportional hazard regressions were used to estimate the hazard ratios (HRs) and 95% confidence intervals (CIs) for the risk of incident CVD. Three models were constricted progressively. Model 1 was adjusted for age and sex; model 2 was further adjusted for education, income, smoking status, drinking status, and physical activity; and model 3 was further adjusted for history of hypertension, diabetes, dyslipidemia, total cholesterol, eGFR, and hs-CRP. The proportional hazards assumption was satisfied by checking the Schoenfeld residual plots. Restricted cubic splines adjusted for variables in model 3 were performed to capture the dose–response relationships of cumMETS-IR and METS-IR slope with the risk of CVD, with 4 knots at the 5th, 35th, 65th, and 95th percentiles of the distribution according to Bayesian information criterion and Akaike information criterion.

Several sensitivity analyses were performed to validate the robustness of the results. First, the competing risk model was performed by considering non-CVD death as a competing risk. Second, to minimize the potential impact of reverse causality, we repeated the primary analysis using a 1 year lagged period by excluding participants who developed CVD cases within the first 1 years of follow-up. Third, restricted analysis was performed by excluding participants with abnormal BMI (≥ 24 kg/m^2^), FBG (≥ 126 mg/dL), TG (≥ 150 mg/dL), and HDL-C (< 38.66 mg/dL). Additionally, subgroup analyses stratified by age (< 60 years vs ≥ 60 years), sex (women vs men), BMI (< 24 kg/m^2^ vs ≥ 24 kg/m^2^), FBG (< 126 mg/dL vs ≥ 126 mg/dL), TG (< 150 mg/dL vs ≥ 150 mg/dL), and HDL-C (< 38.66 mg/dL vs ≥ 38.66 mg/dL) were performed, interaction between subgroups were tested using likelihood ratio tests, in which models with and without multiplicative interaction terms were compared.

All analyses were performed using SAS version 9.4 (SAS Institute, Cary, NC, USA). All the statistical tests were 2-sided, and *P* < 0.05 was considered statistical significance.

## Results

### Baseline characteristics

A comparison in baseline characteristics between excluded and included participants is presented in Additional file 1: Table S1. The mean age of the 47,270 enrolled participants was 48.87 ± 11.77 years, and 36,376 (76.95%) were men. Baseline characteristics according to quartiles of cumMETS-IR are presented in Table 1. Compared with participants in the Q1 group, those with a higher level of cumMETS-IR were more likely to be older, men, less-educated, have a higher prevalence of hypertension, dyslipidemia, more likely to take antihypertensive agents, antidiabetic agents, lipid-lowering agents, and have a higher level of BMI, blood pressure, lipid profiles, hs-CRP and a lower level of eGFR.

**Table 1:** Baseline characteristic of the participants according to quartiles of cumulative METS-IR

Characteristics	Overall	Cumulative METS-IR				*P* value
		Q1 (< 124.84)	Q2 (124.84–142.78)	Q3 (142.79–163.56)	Q4 (≥ 163.57)	
No. of participants	47,270	11,817	11,818	11,818	11,817	
Age, years	48.87 ± 11.77	45.74 ± 11.58	48.31 ± 11.37	49.84 ± 11.65	51.58 ± 11.71	< 0.0001
Men, n (%)	36,376 (77.00)	8307 (70.30)	9256 (78.32)	9430 (79.79)	9383 (79.40)	< 0.0001
High school or above, n (%)	3461 (7.60)	1035 (9.04)	753 (6.60)	823 (7.24)	850 (7.49)	< 0.0001
Income ≥ 800yuan/month, n (%)	6730 (14.80)	1543 (13.49)	1574 (13.82)	1720 (15.15)	1893 (16.68)	< 0.0001
Current smoker, n (%)	15,767 (34.30)	3979 (34.50)	3948 (34.37)	3999 (34.90)	3841 (33.58)	0.1961
Current alcohol, n (%)	18,113 (39.40)	4423 (38.35)	4484 (39.03)	4610 (40.23)	4596 (40.13)	0.0082
Active physical activity, n (%)	6281 (13.30)	1112 (9.41)	1374 (11.63)	1656 (14.01)	2139 (18.10)	< 0.0001
Hypertension, n (%)	18,454 (39.00)	2917 (24.68)	4349 (36.80)	5136 (43.46)	6052 (51.21)	< 0.0001
Diabetes mellitus, n (%)	3749 (7.90)	331 (2.80)	649 (5.49)	1031 (8.72)	1738 (14.71)	< 0.0001
Dyslipidemia, n (%)	16,073 (34.00)	2082 (17.62)	3251 (27.51)	4582 (38.77)	6158 (52.11)	< 0.0001
Antihypertensive agents, n (%)	3842 (8.10)	342 (2.89)	624 (5.28)	1015 (8.59)	1861 (15.75)	< 0.0001
Hypoglycemic agents, n (%)	856 (1.80)	67 (0.57)	124 (1.05)	206 (1.74)	459 (3.88)	< 0.0001
Lipid-lowering agents, n (%)	344 (0.70)	28 (0.24)	49 (0.41)	93 (0.79)	174 (1.47)	< 0.0001
Body mass index, kg/m^2^	25.06 ± 3.47	21.98 ± 2.32	24.31 ± 2.43	25.94 ± 2.64	28.03 ± 3.20	< 0.0001
Systolic blood pressure, mmHg	128.30 ± 19.69	121.54 ± 18.13	127.33 ± 19.07	130.31 ± 19.08	133.99 ± 20.29	< 0.0001
Diastolic blood pressure, mmHg	82.62 ± 11.33	78.90 ± 10.66	82.16 ± 10.99	83.81 ± 10.88	85.59 ± 11.69	< 0.0001
Fasting blood glucose, mg/dL	97.06 ± 27.34	90.70 ± 18.03	94.77 ± 23.73	98.34 ± 28.61	104.44 ± 34.37	< 0.0001
Total cholesterol, mg/dL	189.76 ± 43.88	186.68 ± 39.49	188.35 ± 45.24	189.78 ± 46.56	194.21 ± 43.55	< 0.0001
Triglyceride, mg/dL	149.37 ± 121.9	100.98 ± 74.27	133.6 ± 100.94	161.84 ± 121.72	201.08 ± 153.30	< 0.0001
LDL cholesterol, mg/dL	88.92 ± 35.29	85.56 ± 34.86	89.27 ± 34.37	90.71 ± 35.42	90.17 ± 36.27	< 0.0001
HDL cholesterol, mg/dL	59.99 ± 15.25	64.47 ± 15.17	61.27 ± 14.95	58.87 ± 14.98	55.33 ± 14.39	< 0.0001
eGFR, mL/min/1.73m^2^	84.33 ± 25.12	87.69 ± 28.16	84.15 ± 24.76	82.97 ± 23.92	82.52 ± 23.02	< 0.0001
hs-CRP, mg/L	2.28 ± 6.54	1.95 ± 5.17	2.09 ± 4.99	2.40 ± 8.71	2.69 ± 6.55	< 0.0001

*eGFR* estimated glomerular filtration rate, *LDL* low density lipoprotein, *HDL* high density lipoprotein, *hs-CRP* high-sensitivity C-reactive protein, *METS-IR* metabolic score for insulin resistance

### Cumulative exposure of METS-IR and incident CVD

During a median follow-up of 10.99 years (interquartile range, 10.52–11.32 years), a total of 3,184 cases (6.74%) of incident CVD occurred, including 2,614 cases (5.53%) of stroke and 626 cases (1.32%) of MI. The incidence rate of CVD increased substantially with increasing cumMETS-IR, ranging from 3.91 (95% CI 3.58–4.27) per 1000 person-years in the Q1 group to 9.30 (95% CI 8.77–9.87) per 1000 person-years in the Q4 group, which was also illustrated in Additional file 1: Figure S1 by Kaplan–Meier curves (log-rank *P* < 0.0001). This trend remained significant even after adjustment for potential variables, the HR for the risk of incident CVD was 1.38 (95% CI 1.23–1.54), 1.44 (95% CI 1.29–1.61), and 1.77 (95% CI 1.58–1.98) for the Q2, Q3, and Q4 versus the Q1 group of cumMETS-IR (Table 2). Moreover, there was a linear relationship between cumMETS-IR and the risk of CVD, per 1 standard deviation increase in cumMETS-IR was associated with an 8% higher risk of CVD (HR, 1.08; 95% CI 1.06–1.10).

**Table 2:** Association of cumulative exposure to METS-IR with the risk of cardiovascular disease

Exposure	Case, n (%)	Incidence rate*	Model 1	Model 2	Model 3
Cumulative exposure					
Q1 (n = 11,817)	494 (4.18)	3.91(3.58–4.27)	Reference	Reference	Reference
Q2 (n = 11,818)	757 (6.41)	6.14(5.72–6.59)	1.39(1.24–1.56)	1.40(1.25–1.57)	1.38(1.23–1.54)
Q3 (n = 11,818)	843 (7.13)	6.95(6.49–7.43)	1.47(1.31–1.64)	1.49(1.33–1.66)	1.44(1.29–1.61)
Q4 (n = 11,817)	1090 (9.22)	9.30(8.77–9.87)	1.86(1.67–2.07)	1.90(1.71–2.12)	1.77(1.58–1.98)
*P* for trend			< 0.0001	< 0.0001	< 0.0001
Exposure duration					
0 year (n = 14,380)	500 (4.53)	4.33(3.96–4.72)	Reference	Reference	Reference
2 years (n = 7378)	363 (5.49)	5.28(4.77–5.85)	1.18(1.03–1.35)	1.18(1.03–1.35)	1.17(1.02–1.34)
4 years (n = 7896)	581 (7.49)	7.29(6.72–7.91)	1.56(1.38–1.75)	1.56(1.38–1.76)	1.51(1.34–1.70)
6 years (n = 17,616)	1740 (7.96)	7.76(7.41–8.14)	1.69(1.53–1.86)	1.70(1.54–1.88)	1.60(1.45–1.77)
*P* for trend			< 0.0001	< 0.0001	< 0.0001
Cumulative burden					
< 0 (n = 21,383)	833 (4.86)	4.66(4.35–4.98)	Reference	Reference	Reference
≥ 0 (n = 25,887)	2351 (7.80)	7.60(7.30–7.92)	1.55(1.43–1.68)	1.56(1.44–1.69)	1.49(1.37–1.61)

*METS-IR* metabolic score for insulin resistance

Model 1: adjusted for age and sex;

Model 2: further adjusted for education, income, smoking status, drinking status, and physical activity;

Model 3: further adjusted for history of hypertension, diabetes, dyslipidemia, total cholesterol, estimated glomerular filtration rate, and high sensitivity C-reactive protein

^a^Incidence rate per 1000 person-years

**Fig. 2:**
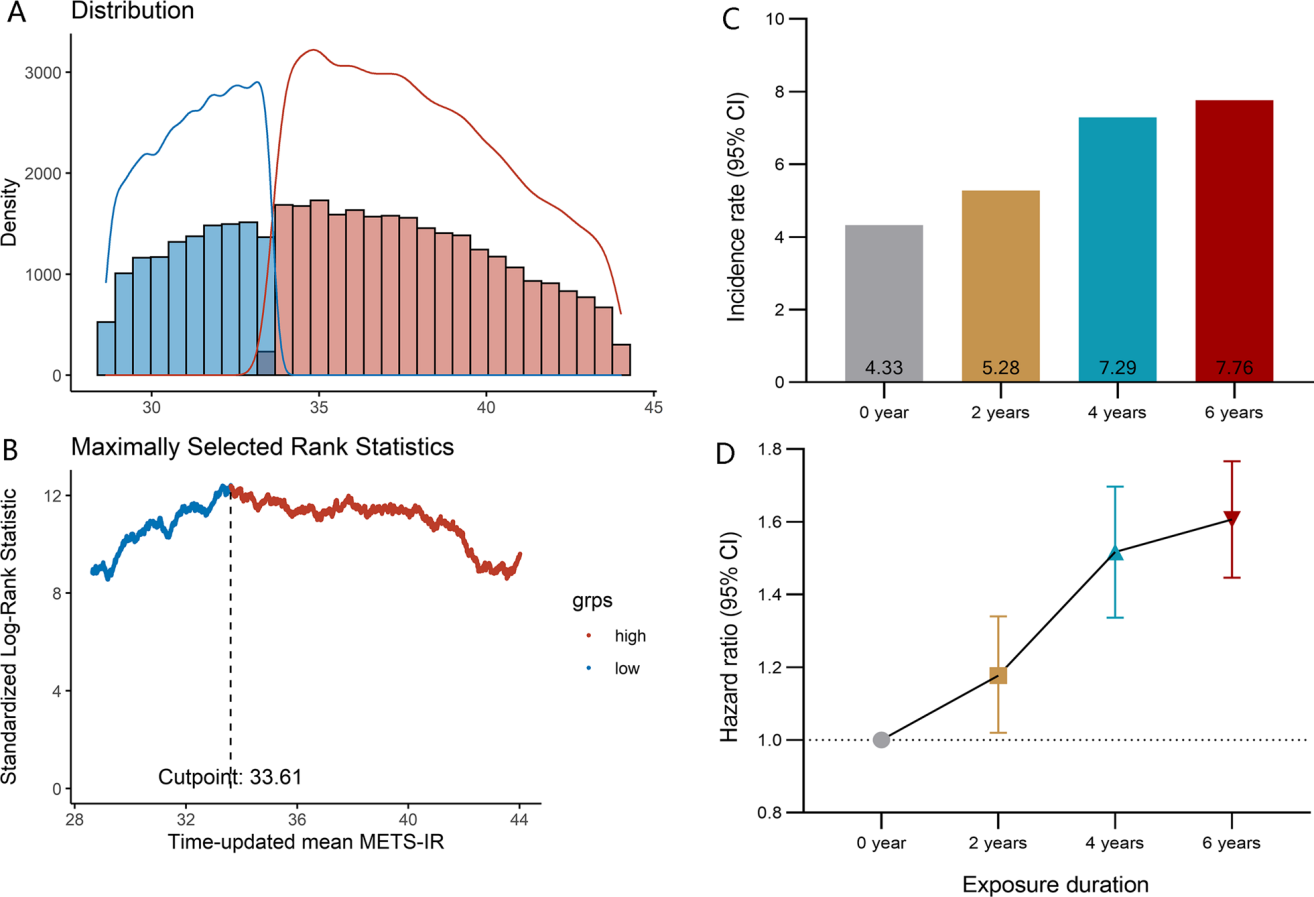
Determining cut-off values of time-weight mean METS-IR with distribution and standardized log-rank statistics (left panel), and the incidence of cardiovascular disease according to exposure duration of METS-IR defined by the cut-off values. A. Plots of the distribution time-updated mean METS-IR B. Standardized log-rank statistical C. Incidence rate of cardiovascular disease according to high METS-IR exposure duration D. Hazard ratio and 95% confidence interval for the association of high METS-IR exposure duration with the risk of cardiovascular disease *CI* confidence interval, *cumMETS-IR* cumulative metabolic score of insulin resistance Adjusted for age, sex, education, income, smoking status, drinking status, physical activity, salt intake, history of hypertension, diabetes, dyslipidemia, total cholesterol, estimated glomerular filtration rate, and high sensitivity C-reactive protein

Using an outcome-oriented method to maximize log-rank statistics, the optimal cutoff point of mean METS-IR associated with CVD was ≥ 33.61 (Fig. 2). With this cutoff, the risk of incident CVD increased when the exposure duration of high METS and cumulative burden increased. Participants with the longest exposure duration of high METS had a 60% higher risk of CVD (adjusted HR, 1.60; 95% CI 1.45–1.77; Table 2 and Fig. 2), and those with cumulative burden over 0 had a 49% higher risk of CVD (HR, 1.49; 95% CI 1.37–1.61; Table 2), compared with their counterparts. The significant associations persisted for incident stroke and MI (Additional file 1: Tables S2, S3; Figures S2, S3).

### Time course of METS-IR accumulation and incident CVD

**Fig. 3:**
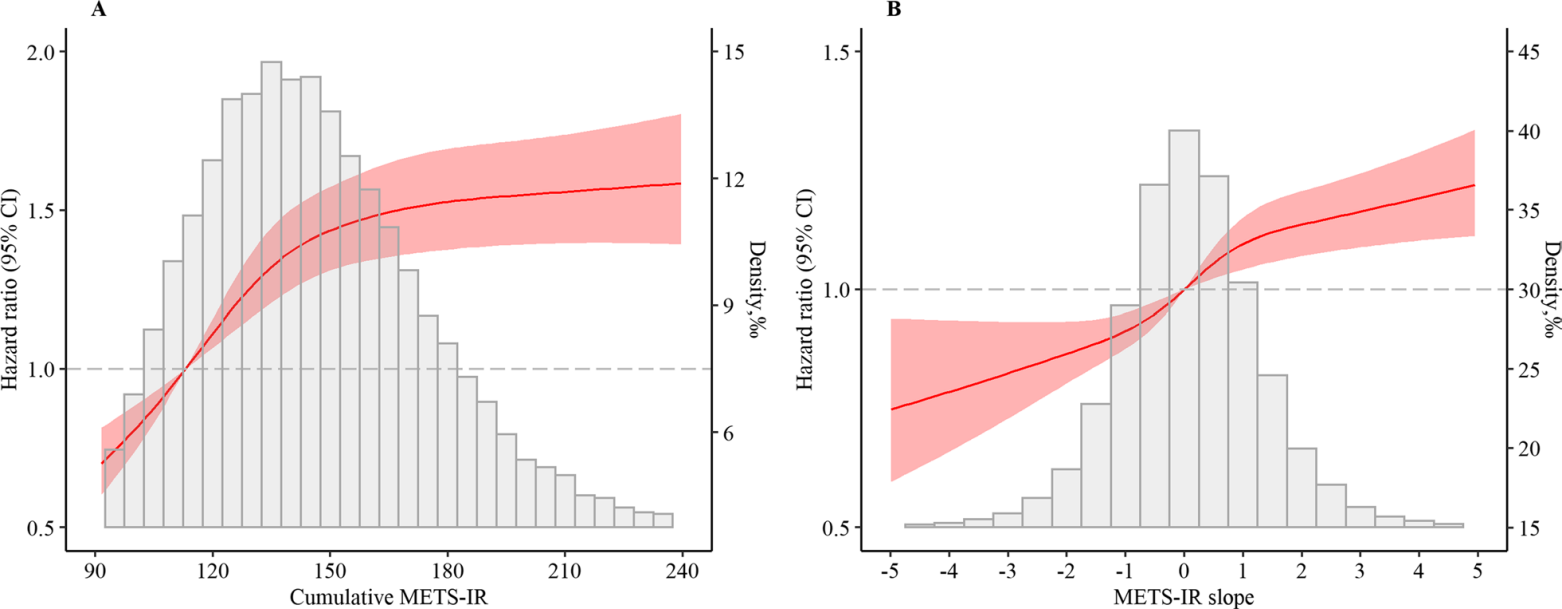
Hazard ratios and 95% CIs for the association of cumMETS-IR and METS-IR slope with the risk of cardiovascular disease by using restricted cubic spline regression with 4 knots with placed at the 5th, 35th, 65th, and 95th percentiles. *CI* confidence interval, *cumMETS-IR* cumulative metabolic score of insulin resistance, *METS-IR* metabolic score of insulin resistance Adjusted for age, sex, education, income, smoking status, drinking status, physical activity, salt intake, history of hypertension, diabetes, dyslipidemia, total cholesterol, estimated glomerular filtration rate, and high sensitivity C-reactive protein

Associations of the time course of METS-IR accumulation with the risk of incident CVD were presented in Table 3. The risk of incident CVD increased with increasing slope of METS-IR (Fig. 3). Participants with a positive slope of METS-IR time course had a 14% higher risk of CVD than those with a negative slope (adjusted HR, 1.14; 95% CI 1.07–1.22). When considering different change patterns, decrease-increase (HR, 1.11; 95% CI 1.00–1.24) and increase-increase (HR, 1.23; 95% CI 1.09–1.38) patterns were associated with higher risk of CVD. When combining cumMETS-IR and slope, participants with CumMETS-IR ≥ median, slope ≥ 0 conferred the highest risk of CVD (HR, 1.38; 95% CI 1.24–1.53). The results were also observed for stroke and MI (Additional file 1: Tables S4, S5, Figures S2, S3).

**Table 3:** Association of time course of cumulative METS-IR with the risk of cardiovascular disease

Exposure	Case, n (%)	Incidence rate^a^	Model 1	Model 2	Model 3
Slope					
< 0 (n = 22,444)	1488 (6.63)	6.43(6.11–6.76)	Reference	Reference	Reference
≥ 0 (n = 24,826)	1696 (6.83)	6.61(6.30–6.93)	1.18(1.10–1.27)	1.17(1.09–1.26)	1.14(1.07–1.22)
Time course patterns					
Decrease-decrease	536 (6.69)	6.48(5.95–7.05)	Reference	Reference	Reference
Decrease-increase	1010 (6.75)	6.50(6.11–6.91)	1.13(1.02–1.26)	1.11(1.00–1.24)	1.11(1.00–1.24)
Increase–decrease	967 (6.56)	6.38(5.99–6.80)	1.08(0.97–1.20)	1.08(0.97–1.20)	1.07(0.96–1.19)
Increase-increase	671 (7.03)	6.81(6.31–7.35)	1.27(1.13–1.43)	1.25(1.11–1.41)	1.23(1.09–1.38)
Combination cumulative exposure and time course					
CumMETS-IR < median, slope < 0	595 (5.13)	4.87(4.49–5.28)	Reference	Reference	Reference
CumMETS-IR < median, slope ≥ 0	656 (5.45)	5.15(4.77–5.56)	1.09(0.97–1.21)	1.08(0.96–1.20)	1.07(0.96–1.20)
CumMETS-IR ≥ median, slope < 0	893 (8.23)	8.17(7.65–8.72)	1.43(1.28–1.58)	1.45(1.31–1.61)	1.38(1.24–1.53)
CumMETS-IR ≥ median, slope ≥ 0	1040 (8.13)	8.05(7.58–8.56)	1.45(1.31–1.60)	1.45(1.31–1.61)	1.38(1.25–1.53)

Model 1: adjusted for age and sex;

Model 2: further adjusted for education, income, smoking status, drinking status, and physical activity;

Model 3: further adjusted for history of hypertension, diabetes, dyslipidemia, total cholesterol, estimated glomerular filtration rate, and high sensitivity C-reactive protein

*METS-IR* metabolic score for insulin resistance

^a^Incidence rate per 1000 person-years

### Additional analyses

Sensitivity analysis with competing risk, excluding incident CVD within 1 year, and restricting participants with normal BMI, FBG, TG, and HDL-C yielded similar results (Additional file 1: Tables S6, S7). Subgroup analyses showed that the associations of cumMETS-IR and time course of METS-IR accumulation with the risk of incident CVD were consistent across the subgroups. There was no significant interaction between cumMETS-IR and time course of METS-IR accumulation and the stratified variables (*P* > 0.05 for interaction; Additional file 1: Tables S8, S9).

## Discussion

This study showed that the risk of future incident CVD was associated with cumMETS-IR exposure and the time course of METS-IR accumulation. Notably, our data suggested that higher cumMETS-IR with an increasing trend in the observation period conferred a greater risk of incident CVD. The risk of CVD prolonged with the exposure duration of high METS-IR increased. Additionally, increased METS-IR, even the METS-IR has decreased trend afterwards could not reverse the risk of CVD acquired by high METS-IR exposure. These findings emphasized the importance of control METS-IR levels to an optimal level across the life course.

The METS-IR score, a novel non-insulin index calculated based on conventional clinical indicators of FBG, TG, HDL-C, and BMI, has been used to screen for early insulin sensitivity and metabolism-illnesses [10]. Since serum insulin levels is not routinely measured in the general clinical field, METS-IR can be applied more easily than insulin-based indexes. Previous studies, which were generally based on a single METS-IR assessment, have investigated the predicting role of METS-IR in the development of cardiovascular events in specific populations [13, 16, 26]. One prospective cohort study with 6,489 Chinese showed that elevated METS-IR was independently associated with incident chronic heart disease, especially in females [26]. Results from the third National Health and Nutrition Examination Survey with 6,043 individuals showed that significantly non-linear association between METS-IR and subclinical MI, especially in in non-diabetic individuals [13]. Similarly, data from a Korean community study showed that a higher METS-IR precedes further ischemic heart disease among 17,943 non-diabetic subjects [16]. A retrospective cohort study with 2031 patients from the Urumqi Research on Sleep Apnea and Hypertension study showed that METS-IR was a powerful predictor of CVD and its subtypes in patients with hypertension and OSA [27]. Consistently, another study found a significant relationship between METS-IR with the risk of stroke among 14,032 hospitalized patients with hypertension [28]. Although the above studies indicated that METS-IR may help identify subjects at high risk of cardiovascular events, the studies are limited by the relatively small sample sizes, a single measurement of METS-IR, and the evident differences in study design and population characteristics, the results warrant confirmation in larger study with repeated measurements.

To our knowledge, this is the first large-scale analysis to examine the long-term effects of cumulative exposure to METS-IR on the risk of CVD. Incorporation of both exposure intensity and duration into one single parameter, as done previously [29–31], our study showed that a higher cumulative exposure, a longer exposure duration, and a higher cumulative burden of METS-IR over 6-years period were all significantly associated with the future risk of CVD, as well as its subtypes of stroke and MI. The results were consistent with aforementioned researches, and confirmed the role of longitudinally dynamic METS-IR in the development of CVD. Additionally, the results were supported by findings on the associations of cumulative exposure to other IR indices with the risk of CVD [31]. Taken together, the findings suggested that METS-IR, as an economic and convenient index of IR, may be used identifying individuals at high risk of developing CVD. In terms of clinical applications, contemporary electronic medical records have potential to automatically calculated cumMETS-IR in order to better stratify high risk population. a time-weighted METS-IR over 33.61 may alter people to establish early lifestyle changes that can reduce atherosclerotic progression.

Furthermore, our study also showed that the risk of CVD also depended on the time course of METS-IR accumulation. Specially, a positive slope (an increasing trend) of METS-IR over time conferred a higher risk of CVD. Individuals with increasing trend, even the levels of METS-IR decreased afterwards, still had a higher risk of CVD. Additionally, incorporation of both cumMETS-IR and the time course, the results showed that same cumMETS-IR exposure accumulated with an increasing trend compared with a decreasing trend contributed more to the subsequent risk of CVD. The results indicated that an increasing in METS-IR over time, even from a relatively lower level to result in the same accumulation, did not fully decrease the risk acquired progressively. Possible reasons may be that atherosclerosis caused by increasing METS-IR, is a chronic progressive disease that begins early in life and develops over the course of decades before becoming clinical manifestation [32].

Although the precise mechanisms linking cumMETS-IR and CVD risk remain incompletely understood, several potential interpretations have been proposed. First, due to the involvement of BMI, METS-IR might be a better indicator of IR in adipose tissue, muscle and liver [33]. Therefore, it can be postulated that an increase in METS-IR over time may reflect IR affecting adipose tissue, muscle and liver. Cumulative IR accelerated the progression of atherosclerosis by altering risk factors and disrupting metabolism through oxidative stress and inflammation [34–36]. Inflammations caused by high cumulative IR could promote the pathophysiological processes of vascular endothelial cells, smooth muscle cells, and macrophages were promoted, which then enhance the formation of atherosclerosis-associated foam cells and vulnerable plaques [37]. Second, cumulative IR was associated with greater platelet adhesion, activation, and aggregation, which leaded to the occlusion of arteries, causing hemodynamic disturbances [38, 39]. Finally, our study showed that participants with high cumMETS-IR coexist with more cardiovascular risk factors, such as higher BMI, blood pressure, lipid profiles, and inflammatory levels, which may also contribute to the progression of CVD.

There are some interesting implications of this study, especially when viewed in the context of other studies. First of all, the assessment of CVD risk is informed by considering not just the total amount of METS-IR, but also the time course of the accumulation. In current practice, the METS-IR at the time is used without trying to incorporate the modulation of that risk by the time course of the individual’s METS-IR levels. We developed a risk model that took into account both of these descriptors of longitudinal METS-IR exposure. The results demonstrated here both emphasized the dependence of risk of CVD, not just on the present METS-IR levels, but also the time course of accumulation, and offer a model to quantify the modulation of risk by the time course. the clinical application of METS-IR. These data suggested that prolonged exposure to lower METS-IR, beginning early, is contributed more to the risk reduction of CVD.

The strengths of our study included the large sample size with a long follow-up, and the components of METS-IR were measured repeatedly. Additionally, we used cumulative value of METS-IR to capture the longitudinal exposure of METS-IR, and incorporated both cumulative exposure and time course of METS-IR accumulation into one risk parameter to predict future CVD, which conferred additional information beyond a single measurement of METS-IR. However, several limitations should also be noted. First, insulin concentrations were not collected in our study due to the large population with high cost, we could not compare the predict value of cumMETS-IR with the cumulation of the gold-standard for the risk of CVD. Second, owing to the observational nature of the study, we could not establish a causal association of cumMETS-IR with the risk of CVD. Third, residual confounding cannot be completely ruled out due to the limitation of observational study design, despite comprehensive adjustment for the potential confounders. Finally, the sex distribution of the sample was unbalanced. However, the associations were statistically robust, given that a significant interaction was not identified when data were stratified by sex.

## Conclusions

Incident CVD risk was associated with both long-term exposure to METS-IR and the time course of METS-IR accumulation. Importantly, the same cumMETS-IR with an increasing trend resulted in a greater risk increase, emphasizing the importance of control an optimal METS-IR across the lifespan.

### Supplementary Information

**Additional file 1: Table S1.** Baseline characteristics of excluded and included participants. **Table S2.** Association of cumulative exposure to METS-IR with the risk of stroke. **Table S3.** Association of cumulative exposure to METS-IR with the risk of myocardial infarction. **Table S4.** Association of time course of cumulative METS-IR with the risk of stroke. **Table S5.** Association of time course of cumulative METS-IR with the risk of myocardial infarction. **Table S6.** Sensitivity analyses for the association of cumulative METS-IR with the risk of cardiovascular disease. **Table S7.** Sensitivity analyses for the association of time course of cumulative METS-IR with the risk of cardiovascular disease. **Table S8.** Subgroup analyses for the association of cumulative METS-IR with the risk of cardiovascular disease. **Table S9.** Subgroup analyses for the association of time course of cumulative METS-IR with the risk of cardiovascular disease. **Figure S1.** Kaplan-Meier curves of cardiovascular disease and its subtypes incidence rate by quartiles of cumulative exposure to metabolic score of insulin resistance. **Figure S2.** Hazard ratios and 95% CIs for the association of cumMETS-IR and METS-IR slope with the risk of stroke by using restricted cubic spline regression with 4 knots with placed at the 5th, 35th, 65th, and 95th percentiles. **Figure S3.** Hazard ratios and 95% CIs for the association of cumMETS-IR and METS-IR slope with the risk of myocardial infarction by using restricted cubic spline regression with 4 knots with placed at the 5th, 35th, 65th, and 95th percentiles.

## Data Availability

Data are available to researchers on request for purposes of reproducing the results or replicating the procedure by directly contacting the corresponding author.
