# Supplementary material for: Magnitude and time course of insulin resistance accumulation with the risk of cardiovascular disease: an 11-years cohort study
Source: Cardiovasc Diabetol. 2023 Dec 13;22:339. doi: 10.1186/s12933-023-02073-2 (PMC10720129; doi:10.1186/s12933-023-02073-2)
Supplement: Supplementary file 1 — Additional file 1: Table S1. Baseline characteristics of excluded and included participants. Table S2. Association of cumulative exposure to METS-IR with the risk of stroke. Table S3. Association of cumulative exposure to METS-IR with the risk of myocardial infarction. Table S4. Association of time course of cumulative METS-IR with the risk of stroke. Table S5. Association of time course of cumulative METS-IR with the risk of myocardial infarction. Table S6. Sensitivity analyses for the association of cumulative METS-IR with the risk of cardiovascular disease. Table S7. Sensitivity analyses for the association of time course of cumulative METS-IR with the risk of cardiovascular disease. Table S8. Subgroup analyses for the association of cumulative METS-IR with the risk of cardiovascular disease. Table S9. Subgroup analyses for the association of time course of cumulative METS-IR with the risk of cardiovascular disease. Figure S1. Kaplan-Meier curves of cardiovascular disease and its subtypes incidence rate by quartiles of cumulative exposure to metabolic score of insulin resistance. Figure S2. Hazard ratios and 95% CIs for the association of cumMETS-IR and METS-IR slope with the risk of stroke by using restricted cubic spline regression with 4 knots with placed at the 5th, 35th, 65th, and 95th percentiles. Figure S3. Hazard ratios and 95% CIs for the association of cumMETS-IR and METS-IR slope with the risk of myocardial infarction by using restricted cubic spline regression with 4 knots with placed at the 5th, 35th, 65th, and 95th percentiles. [file 12933_2023_2073_MOESM1_ESM.docx]

**Additional file Materials**

Table S1. Baseline characteristics of excluded and included participants

| Characteristics | Excluded  (n=54240) | Included  (n=47270) | *P* value |
| --- | --- | --- | --- |
| Age, years | 54.61±12.81 | 48.87±11.77 | <0.0001 |
| Men, n (%) | 44734 (82.47) | 36376 (76.95) | <0.0001 |
| High school or above, n (%) | 3309 (6.37) | 3461 (7.60) | <0.0001 |
| Income≥800yuan/month, n (%) | 7285 (14.03) | 6730 (14.78) | 0.0010 |
| Current smoker, n (%) | 18028 (34.42) | 15767 (34.34) | 0.7860 |
| Current alcohol, n (%) | 18539 (35.39) | 18113 (39.43) | <0.0001 |
| Active physical activity, n (%) | 9000 (16.59) | 6281 (13.29) | <0.0001 |
| Hypertension, n (%) | 26199 (48.30) | 18454 (39.04) | <0.0001 |
| Diabetes mellitus, n (%) | 5740 (10.58) | 3749 (7.93) | <0.0001 |
| Dyslipidemia, n (%) | 20320 (37.46) | 16073 (34.00) | <0.0001 |
| Antihypertensive agents, n (%) | 7472 (13.78) | 3842 (8.13) | <0.0001 |
| Hypoglycemic agents, n (%) | 1625 (3.00) | 856 (1.81) | <0.0001 |
| Lipid-lowering agents, n (%) | 619 (1.14) | 344 (0.73) | <0.0001 |
| Body mass index, kg/m^2^ | 25.02±3.52 | 25.06±3.47 | 0.0759 |
| Systolic blood pressure, mmHg | 133.53±21.94 | 128.3±19.69 | <0.0001 |
| Diastolic blood pressure, mmHg | 84.26±12.12 | 82.62±11.33 | <0.0001 |
| Fasting blood glucose, mg/dL | 100.03±32.75 | 97.06±27.34 | <0.0001 |
| Total cholesterol, mg/dL | 192.82±44.87 | 189.76±43.88 | <0.0001 |
| Triglyceride, mg/dL | 147.74±121.39 | 149.37±121.9 | 0.0334 |
| LDL cholesterol, mg/dL | 92.36±35.27 | 88.92±35.29 | <0.0001 |
| HDL cholesterol, mg/dL | 59.63±15.91 | 59.99±15.25 | 0.0003 |
| eGFR, mL/min/1.73m^2^ | 79.77±25.91 | 84.33±25.12 | <0.0001 |
| hs-CRP, mg/L | 2.52±6.4 | 2.28±6.54 | <0.0001 |

Abbreviations: eGFR, estimated glomerular filtration rate; LDL, low density lipoprotein; HDL, high density lipoprotein; hs-CRP, high-sensitivity C-reactive protein.

Table S2. Association of cumulative exposure to METS-IR with the risk of stroke

| Exposure | Case, n (%) | Incidence rate* | Model 1 | Model 2 | Model 3 |
| --- | --- | --- | --- | --- | --- |
| Cumulative exposure |  |  |  |  |  |
| Q1 (n=11817) | 413 (3.49) | 3.26(2.96-3.59) | Reference | Reference | Reference |
| Q2 (n=11818) | 621 (5.25) | 5.01(4.63-5.42) | 1.36(1.20-1.54) | 1.37(1.21-1.55) | 1.35(1.19-1.53) |
| Q3 (n=11818) | 707 (5.98) | 5.79(5.38-6.24) | 1.47(1.30-1.66) | 1.49(1.32-1.69) | 1.45(1.28-1.64) |
| Q4 (n=11817) | 873 (7.39) | 7.38(6.91-7.89) | 1.78(1.58-2.00) | 1.82(1.61-2.05) | 1.70(1.50-1.92) |
| *P* for trend |  |  | <0.0001 | <0.0001 | <0.0001 |
| Exposure duration |  |  |  |  |  |
| 0 year (n=14380) | 420 (3.80) | 3.63(3.30-3.99) | Reference | Reference | Reference |
| 2 years (n=7378) | 306 (4.63) | 4.44(3.97-4.96) | 1.19(1.02-1.37) | 1.18(1.02-1.37) | 1.17(1.01-1.36) |
| 4 years (n=7896) | 481 (6.20) | 6.00(5.48-6.56) | 1.53(1.34-1.74) | 1.53(1.34-1.74) | 1.48(1.30-1.69) |
| 6 years (n=17616) | 1407 (6.44) | 6.23(5.91-6.56) | 1.62(1.45-1.81) | 1.63(1.46-1.82) | 1.54(1.38-1.72) |
| *P* for trend |  |  | <0.0001 | <0.0001 | <0.0001 |
| Cumulative burden |  |  |  |  |  |
| <0 (n=21383) | 700 (4.08) | 3.90(3.62-4.20) | Reference | Reference | Reference |
| ≥0 (25887) | 1914 (6.35) | 6.15(5.88-6.43) | 1.50(1.37-1.63) | 1.51(1.38-1.64) | 1.44(1.32-1.57) |

Abbreviations: METS-IR, metabolic score for insulin resistance

* Incidence rate per 1000 person-years.

Model 1: adjusted for age and sex;

Model 2: further adjusted for education, income, smoking status, drinking status, and physical activity;

Model 3: further adjusted for history of hypertension, diabetes, dyslipidemia, antihypertensive agents, antidiabetic agents, lipid-lowering agents, body mass index, systolic blood pressure, diastolic blood pressure, fasting blood glucose, total cholesterol, estimated glomerular filtration rate, and high sensitivity C-reactive protein.

Table S3. Association of cumulative exposure to METS-IR with the risk of myocardial infarction

| Exposure | Case, n (%) | Incidence rate* | Model 1 | Model 2 | Model 3 |
| --- | --- | --- | --- | --- | --- |
| Cumulative exposure |  |  |  |  |  |
| Q1 (n=11817) | 86 (0.73) | 0.67(0.54-0.83) | Reference | Reference | Reference |
| Q2 (n=11818) | 149 (1.26) | 1.19(1.01-1.39) | 1.55(1.19-2.02) | 1.56(1.19-2.03) | 1.52(1.17-1.98) |
| Q3 (n=11818) | 154 (1.30) | 1.24(1.06-1.45) | 1.50(1.15-1.96) | 1.52(1.17-1.99) | 1.45(1.11-1.89) |
| Q4 (n=11817) | 237 (2.01) | 1.96(1.73-2.23) | 2.25(1.75-2.88) | 2.30(1.79-2.95) | 2.09(1.62-2.70) |
| *P* for trend |  |  | <0.0001 | <0.0001 | <0.0001 |
| Exposure duration |  |  |  |  |  |
| 0 year (n=14380) | 87 (0.79) | 0.74(0.60-0.92) | Reference | Reference | Reference |
| 2 years (n=7378) | 62 (0.94) | 0.89(0.69-1.14) | 1.15(0.83-1.60) | 1.16(0.84-1.60) | 1.14(0.83-1.58) |
| 4 years (n=7896) | 111 (1.43) | 1.36(1.13-1.64) | 1.67(1.26-2.21) | 1.69(1.28-2.23) | 1.61(1.21-2.13) |
| 6 years (n=17616) | 366 (1.67) | 1.59(1.44-1.76) | 1.99(1.57-2.51) | 2.00(1.59-2.53) | 1.85(1.46-2.34) |
| *P* for trend |  |  | <0.0001 | <0.0001 | <0.0001 |
| Cumulative burden |  |  |  |  |  |
| <0 (n=21383) | 146 (0.85) | 0.80(0.68-0.95) | Reference | Reference | Reference |
| ≥0 (25887) | 480 (1.59) | 1.51(1.38-1.66) | 1.76(1.47-2.12) | 1.78(1.48-2.14) | 1.66(1.38-2.00) |

Abbreviations: METS-IR, metabolic score for insulin resistance

* Incidence rate per 1000 person-years.

Model 1: adjusted for age and sex;

Model 2: further adjusted for education, income, smoking status, drinking status, and physical activity;

Model 3: further adjusted for history of hypertension, diabetes, dyslipidemia, antihypertensive agents, antidiabetic agents, lipid-lowering agents, body mass index, systolic blood pressure, diastolic blood pressure, fasting blood glucose, total cholesterol, estimated glomerular filtration rate, and high sensitivity C-reactive protein.

Table S4. Association of time course of cumulative METS-IR with the risk of stroke

| Exposure | Case, n (%) | Incidence rate* | Model 1 | Model 2 | Model 3 |
| --- | --- | --- | --- | --- | --- |
| Slope |  |  |  |  |  |
| <0 (n=22444) | 1235 (5.50) | 5.31(5.02-5.61) | Reference | Reference | Reference |
| ≥0 (n=24826) | 1379 (5.55) | 5.34(5.07-5.63 | 1.15(1.06-1.24) | 1.14(1.05-1.23) | 1.12(1.05-1.21) |
| Time course patterns |  |  |  |  |  |
| Decrease-decrease | 448 (5.59) | 4.14(3.80-4.52) | Reference | Reference | Reference |
| Decrease-increase | 821 (5.48) | 4.11(3.77-4.48) | 1.10(0.98-1.23) | 1.08(0.96-1.21) | 1.07(0.96-1.21) |
| Increase-decrease | 806 (5.47) | 6.60(6.14-7.10) | 1.06(0.95-1.20) | 1.07(0.95-1.20) | 1.05(0.93-1.18) |
| Increase-increase | 539 (5.65) | 6.56(6.13-7.01) | 1.20(1.05-1.36) | 1.19(1.04-1.35) | 1.16(1.02-1.32) |
| Combination |  |  |  |  |  |
| CumMETS-IR<median, slope < 0 | 508 (4.38) | 5.39(4.91-5.91) | Reference | Reference | Reference |
| CumMETS-IR<median, slope ≥ 0 | 526 (4.37) | 5.25(4.91-5.62) | 1.02(0.90-1.15) | 1.01(0.89-1.14) | 1.01(0.89-1.14) |
| CumMETS-IR≥ median, slope < 0 | 727 (6.70) | 5.29(4.94-5.67) | 1.36(1.21-1.52) | 1.38(1.23-1.55) | 1.32(1.17-1.48) |
| CumMETS-IR≥ median, slope ≥ 0 | 853 (6.67) | 5.43(4.99-5.91) | 1.39(1.24-1.55) | 1.40(1.25-1.56) | 1.34(1.20-1.50) |

Abbreviations: METS-IR, metabolic score for insulin resistance

* Incidence rate per 1000 person-years.

Model 1: adjusted for age and sex;

Model 2: further adjusted for education, income, smoking status, drinking status, and physical activity;

Model 3: further adjusted for history of hypertension, diabetes, dyslipidemia, total cholesterol, estimated glomerular filtration rate, and high sensitivity C-reactive protein.

Table S5. Association of time course of cumulative METS-IR with the risk of myocardial infarction

| Exposure | Case, n (%) | Incidence rate* | Model 1 | Model 2 | Model 3 |
| --- | --- | --- | --- | --- | --- |
| Slope |  |  |  |  |  |
| <0 (n=22444) | 277 (1.23) | 1.17(1.04-1.32) | Reference | Reference | Reference |
| ≥0 (n=24826) | 349 (1.41) | 1.33(1.20-1.48) | 1.36(1.16-1.60) | 1.33(1.13-1.57) | 1.24(1.07-1.43) |
| Time course patterns |  |  |  |  |  |
| Decrease-decrease | 95 (1.19) | 0.78(0.64-0.95) | Reference | Reference | Reference |
| Decrease-increase | 211 (1.41) | 1.07(0.90-1.26) | 1.36(1.06-1.73) | 1.32(1.03-1.68) | 1.31(1.04-1.69) |
| Increase-decrease | 177 (1.20) | 1.60(1.38-1.85) | 1.17(0.90-1.50) | 1.15(0.89-1.49) | 1.15(0.89-1.48) |
| Increase-increase | 143 (1.50) | 1.59(1.39-1.82) | 1.62(1.24-2.11) | 1.56(1.20-2.04) | 1.54(1.19-2.01) |
| Combination |  |  |  |  |  |
| CumMETS-IR<median, slope < 0 | 97 (0.84) | 1.12(0.92-1.37) | Reference | Reference | Reference |
| CumMETS-IR<median, slope ≥ 0 | 138 (1.15) | 1.33(1.16-1.52) | 1.41(1.08-1.82) | 1.38(1.07-1.79) | 1.37(1.06-1.78) |
| CumMETS-IR≥ median, slope < 0 | 180 (1.66) | 1.14(0.99-1.33) | 1.72(1.35-2.21) | 1.75(1.37-2.25) | 1.64(1.28-2.11) |
| CumMETS-IR≥ median, slope ≥ 0 | 211 (1.65) | 1.42(1.21-1.68) | 1.77(1.39-2.25) | 1.76(1.38-2.24) | 1.63(1.28-2.08) |

Abbreviations: METS-IR, metabolic score for insulin resistance

* Incidence rate per 1000 person-years.

Model 1: adjusted for age and sex;

Model 2: further adjusted for education, income, smoking status, drinking status, and physical activity;

Model 3: further adjusted for history of hypertension, diabetes, dyslipidemia, total cholesterol, estimated glomerular filtration rate, and high sensitivity C-reactive protein.

Table S6. Sensitivity analyses for the association of cumulative METS-IR with the risk of cardiovascular disease

| Outcomes | Quartiles of cumulative METS-IR | | | | *P* for trend |
| --- | --- | --- | --- | --- | --- |
|  | Q1 | Q2 | Q3 | Q4 |  |
| Competing risk model |  |  |  |  |  |
| Cardiovascular disease | Reference | 1.38(1.23-1.54) | 1.45(1.29-1.62) | 1.76(1.58-1.97) | <0.0001 |
| Stroke | Reference | 1.35(1.19-1.53) | 1.46(1.29-1.65) | 1.69(1.50-1.91) | <0.0001 |
| Myocardial infarction | Reference | 1.52(1.16-1.98) | 1.46(1.12-1.90) | 2.08(1.61-2.68) | <0.0001 |
| 1-year lagged analysis (n=46889) | |  |  |  |  |
| Cardiovascular disease | Reference | 1.37(1.22-1.54) | 1.39(1.24-1.56) | 1.75(1.56-1.95) | <0.0001 |
| Stroke | Reference | 1.35(1.19-1.53) | 1.40(1.24-1.59) | 1.70(1.50-1.92) | <0.0001 |
| Myocardial infarction | Reference | 1.48(1.13-1.95) | 1.35(1.02-1.77) | 1.95(1.50-2.54) | <0.0001 |
| Restricted analysis (n=13900)* | |  |  |  |  |
| Cardiovascular disease | Reference | 1.29(1.08-1.53) | 1.31(1.04-1.65) | 1.58(1.13-2.22) | <0.0001 |
| Stroke | Reference | 1.21(0.99-1.47) | 1.29(1.00-1.66) | 1.77(1.25-2.52) | <0.0001 |
| Myocardial infarction | Reference | 1.68(1.11-2.54) | 1.62(0.94-2.79) | 0.76(0.24-2.46) | 0.1817 |

Abbreviations: METS-IR, metabolic score for insulin resistance

*Restricted analysis was performed by restricting patients with BMI<24 kg/m^2^, FBG<126 mg/dL, TG<150mg/dL, and HDL-C≥38.66mg/dL.

Adjusted for age, sex, education, income, smoking status, drinking status, physical activity, history of hypertension, diabetes, dyslipidemia, total cholesterol, estimated glomerular filtration rate, and high sensitivity C-reactive protein.

Table S7. Sensitivity analyses for the association of time course of cumulative METS-IR with the risk of cardiovascular disease

| Outcomes | Time course of cumulative METS-IR | | | |
| --- | --- | --- | --- | --- |
|  | CumMETS-IR<median,  slope < 0 | CumMETS-IR<median,  slope ≥ 0 | CumMETS-IR≥median, slope < 0 | CumMETS-IR≥median, slope ≥ 0 |
| Competing risk model |  |  |  |  |
| Cardiovascular disease | Reference | 1.08(0.97-1.21) | 1.39(1.25-1.54) | 1.39(1.25-1.54) |
| Stroke | Reference | 1.02(0.90-1.15) | 1.33(1.18-1.49) | 1.34(1.20-1.50) |
| Myocardial infarction | Reference | 1.38(1.06-1.79) | 1.65(1.29-2.12) | 1.63(1.28-2.09) |
| 1-year lagged analysis (n=46889) | |  |  |  |
| Cardiovascular disease | Reference | 1.09(0.97-1.22) | 1.36(1.22-1.52) | 1.36(1.23-1.51) |
| Stroke | Reference | 1.01(0.90-1.15) | 1.30(1.16-1.47) | 1.32(1.18-1.48) |
| Myocardial infarction | Reference | 1.45(1.11-1.90) | 1.64(1.26-2.13) | 1.58(1.22-2.03) |
| Restricted analysis (n=13900)* | |  |  |  |
| Cardiovascular disease | Reference | 1.21(1.01-1.45) | 1.25(0.85-1.85) | 1.44(1.13-1.83) |
| Stroke | Reference | 1.07(0.88-1.31) | 1.24(0.82-1.88) | 1.40(1.08-1.80) |
| Myocardial infarction | Reference | 2.25(1.38-3.67) | 1.31(0.44-3.86) | 2.14(1.14-4.00) |

Abbreviations: cumMETS-IR, cumulative metabolic score for insulin resistance

*Restricted analysis was performed by restricting patients with BMI<24 kg/m^2^, FBG<126 mg/dL, TG<150mg/dL, and HDL-C>38.66mg/dL.

Adjusted for age, sex, education, income, smoking status, drinking status, physical activity, history of hypertension, diabetes, dyslipidemia, total cholesterol, estimated glomerular filtration rate, and high sensitivity C-reactive protein.

Table S8. Subgroup analyses for the association of cumulative METS-IR with the risk of cardiovascular disease

| Outcomes | Quartiles of cumulative METS-IR | | | | *P* for interaction |  |  |  |  |
| --- | --- | --- | --- | --- | --- | --- | --- | --- | --- |
|  | Q1 | Q2 | Q3 | Q4 |  |  |  |  |  |
| Age |  |  |  |  |  |  |  |  |  |
| <60 years | Reference | 1.35(1.19-1.54) | 1.43(1.25-1.63) | 1.78(1.57-2.02) | 0.1827 |  |  |  |  |
| ≥60 years | Reference | 1.32(1.05-1.66) | 1.30(1.04-1.62) | 1.49(1.21-1.85) |  |  |  |  |  |
| Sex |  |  |  |  |  |  |  |  |  |
| Women | Reference | 1.45(1.04-2.01) | 1.64(1.19-2.26) | 1.84(1.34-2.51) | 0.4713 |  |  |  |  |
| Men | Reference | 1.36(1.20-1.53) | 1.40(1.24-1.58) | 1.73(1.54-1.94) |  |  |  |  |  |
| Body mass index |  |  |  |  |  |  |  |  |  |
| <24 kg/m^2^ | Reference | 1.32(1.14-1.54) | 1.50(1.26-1.78) | 1.62(1.28-2.06) | 0.1548 |  |  |  |  |
| ≥24 kg/m^2^ | Reference | 1.20(0.97-1.48) | 1.19(0.97-1.46) | 1.50(1.23-1.84) |  |  |  |  |  |
| Fasting blood glucose |  |  |  |  |  |  |  |  |  |
| <126 mg/dL | Reference | 1.36(1.21-1.53) | 1.37(1.22-1.54) | 1.71(1.52-1.91) | 0.0518 |  |  |  |  |
| ≥126 mg/dL | Reference | 1.17(0.76-1.81) | 1.37(0.91-2.06) | 1.32(0.89-1.96) |  |  |  |  |  |
| Triglyceride |  |  |  |  |  |  |  |  |  |
| <150 mg/dL | Reference | 1.36(1.20-1.55) | 1.35(1.18-1.54) | 1.61(1.40-1.85) | 0.8511 |  |  |  |  |
| ≥150 mg/dL | Reference | 1.23(0.95-1.60) | 1.32(1.03-1.69) | 1.55(1.22-1.98) |  |  |  |  |  |
| High density lipoprotein cholesterol | | | | | |  |  |  |  |
| <38.66 mg/dL | Reference | 2.30(0.99-5.35) | 1.83(0.80-4.15) | 3.10(1.42-6.76) | 0.2359 |  |  |  |  |
| ≥38.66 mg/dL | Reference | 1.36(1.21-1.53) | 1.44(1.28-1.61) | 1.72(1.54-1.92) |  |  |  |  |  |

Abbreviations: BMI, body mass index; METS-IR, metabolic score for insulin resistance

Adjusted for age, sex, education, income, smoking status, drinking status, physical activity, history of hypertension, diabetes, dyslipidemia, total cholesterol, estimated glomerular filtration rate, and high sensitivity C-reactive protein other than variables for stratification.

Table S9. Subgroup analyses for the association of time course of cumulative METS-IR with the risk of cardiovascular disease

| Outcomes | CumMETS-IR<median,  slope < 0 | CumMETS-IR<median,  slope ≥ 0 | CumMETS-IR≥median, slope < 0 | CumMETS-IR≥median, slope ≥ 0 | *P* for interaction |  |  |  |  |  |
| --- | --- | --- | --- | --- | --- | --- | --- | --- | --- | --- |
| Age |  |  |  |  |  |  |  |  |  |  |
| <60 years | Reference | 1.08(0.95-1.23) | 1.38(1.22-1.56) | 1.41(1.25-1.59) | 0.3215 |  |  |  |  |  |
| ≥60 years | Reference | 1.07(0.86-1.33) | 1.24(1.02-1.52) | 1.21(1.00-1.48) |  |  |  |  |  |  |
| Sex |  |  |  |  |  |  |  |  |  |  |
| Women | Reference | 0.96(0.70-1.33) | 1.36(1.01-1.84) | 1.41(1.05-1.88) | 0.3426 |  |  |  |  |  |
| Men | Reference | 1.09(0.97-1.23) | 1.36(1.22-1.53) | 1.37(1.23-1.53) |  |  |  |  |  |  |
| Body mass index |  |  |  |  |  |  |  |  |  |  |
| <24 kg/m^2^ | Reference | 1.19(1.03-1.39) | 1.36(1.05-1.76) | 1.55(1.29-1.85) | 0.0520 |  |  |  |  |  |
| ≥24 kg/m^2^ | Reference | 1.06(0.89-1.25) | 1.20(1.05-1.37) | 1.20(1.05-1.37) |  |  |  |  |  |  |
| Fasting blood glucose | | | | | |  |  |  |  |  |
| <126 mg/dL | Reference | 1.09(0.97-1.22) | 1.33(1.18-1.49) | 1.35(1.21-1.50) | 0.2027 |  |  |  |  |  |
| ≥126 mg/dL | Reference | 1.40(0.93-2.10) | 1.28(0.95-1.73) | 1.45(1.07-1.97) |  |  |  |  |  |  |
| Triglyceride |  |  |  |  |  |  |  |  |  |  |
| <150 mg/dL | Reference | 1.11(0.98-1.26) | 1.31(1.14-1.50) | 1.30(1.15-1.48) | 0.8243 |  |  |  |  |  |
| ≥150 mg/dL | Reference | 1.06(0.84-1.34) | 1.24(1.04-1.48) | 1.32(1.11-1.58) |  |  |  |  |  |  |
| High density lipoprotein cholesterol | | | | | |  |  |  |  |  |
| <38.66 mg/dL | Reference | 0.87(0.30-2.50) | 1.22(0.79-1.91) | 2.03(1.24-3.30) | 0.0532 |  |  |  |  |  |
| ≥38.66 mg/dL | Reference | 1.08(0.96-1.20) | 1.39(1.24-1.55) | 1.36(1.22-1.51) |  |  |  |  |  |  |

Abbreviations: BMI, body mass index; METS-IR, metabolic score for insulin resistance

Adjusted for age, sex, education, income, smoking status, drinking status, physical activity, history of hypertension, diabetes, dyslipidemia, total cholesterol, estimated glomerular filtration rate, and high sensitivity C-reactive protein other than variables for stratification.


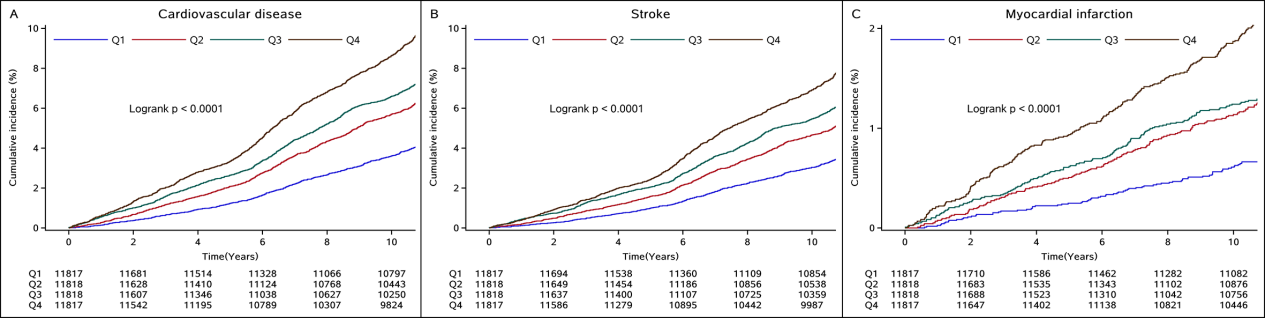


**Figure S1. Kaplan-Meier curves of cardiovascular disease and its subtypes incidence rate by quartiles of cumulative exposure to metabolic score of insulin resistance**


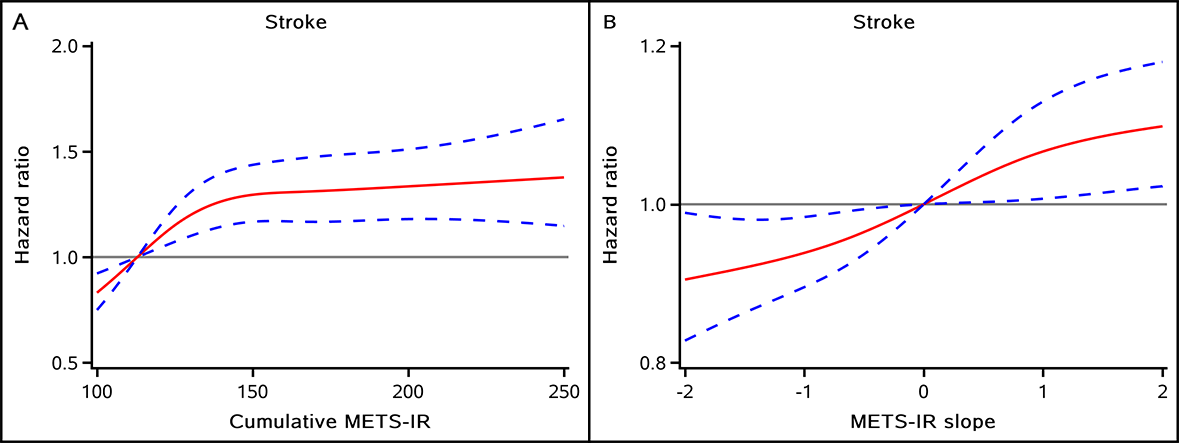


**Figure S2. Hazard ratios and 95% CIs for the association of cumMETS-IR and METS-IR slope with the risk of stroke by using restricted cubic spline regression with 4 knots with placed at the 5th, 35th, 65th, and 95th percentiles.**

Abbreviations: CI, confidence interval; cumMETS-IR, cumulative metabolic score of insulin resistance; METS-IR, metabolic score of insulin resistance

Adjusted for age, sex, education, income, smoking status, drinking status, physical activity, history of hypertension, diabetes, dyslipidemia, total cholesterol, estimated glomerular filtration rate, and high sensitivity C-reactive protein.


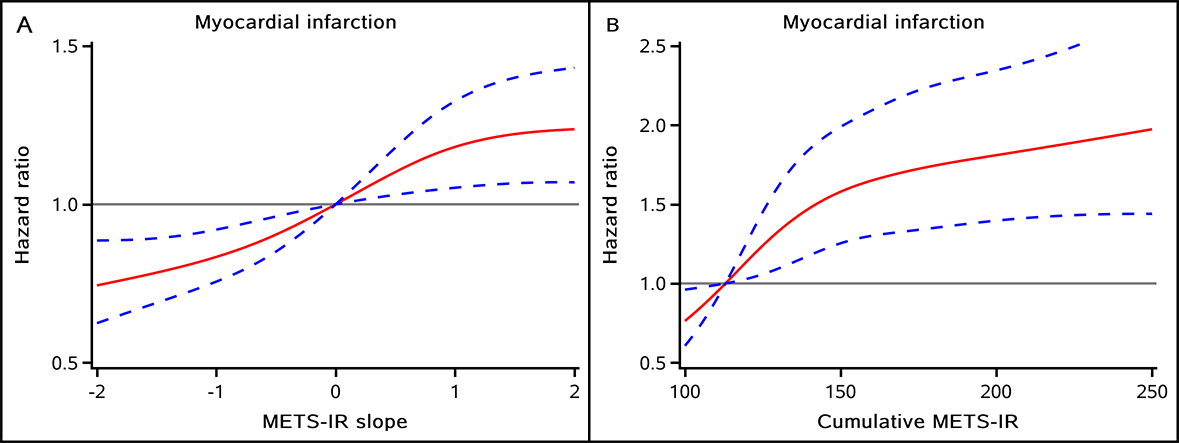


**Figure S3. Hazard ratios and 95% CIs for the association of cumMETS-IR and METS-IR slope with the risk of myocardial infarction by using restricted cubic spline regression with 4 knots with placed at the 5th, 35th, 65th, and 95th percentiles.**

Abbreviations: CI, confidence interval; cumMETS-IR, cumulative metabolic score of insulin resistance; METS-IR, metabolic score of insulin resistance

Adjusted for age, sex, education, income, smoking status, drinking status, physical activity, history of hypertension, diabetes, dyslipidemia, total cholesterol, estimated glomerular filtration rate, and high sensitivity C-reactive protein.
